# Effect of polymerase chain reaction-based antifungal stewardship on candidemia management in small- and medium-sized hospitals

**DOI:** 10.1186/s40780-026-00596-w

**Published:** 2026-06-11

**Authors:** Koji Miyazaki, Tomoyuki Ishigo, Miyu Yamada, Ryoma Takeda, Naoki Wada, Marie Itaya, Masaji Saijo, Takanobu Hoshi, Yoshitaka Saito

**Affiliations:** 1 Department of Pharmacy, Sapporo Tokushukai Hospital, Oyachi-higashi 1-jo 1-chome 1-1, Atsubetsu-ku, Sapporo-shi, Hokkaido 004-0041 Japan; 2 Department of Pharmacy, Sapporo Medical University Hospital, South 1, West 16, Chuo-ku, Sapporo-shi, Hokkaido 060-8543 Japan; 3 Division of Clinical Pharmacy, Department of Pharmacy, Hokkaido University of Science, Maeda 7-jo 15-chome 4-1, Teine-ku, Sapporo-shi, Hokkaido 006-8585 Japan; 4 Department of Pharmacy, Nakamura Memorial Hospital, Minami 1-jo Nishi 14-chome 291-190, Chuo-ku, Sapporo-shi, Hokkaido 060-8570 Japan; 5 Department of Clinical Laboratory, Sapporo Tokushukai Hospital, Oyachi-higashi 1-jo 1-chome 1-1, Atsubetsu-ku, Sapporo-shi, Hokkaido 004-0041 Japan; 6 Department of Primary Care, Sapporo Tokushukai Hospital, Oyachi-higashi 1-jo 1- chome 1-1, Atsubetsu-ku, Sapporo-shi, Hokkaido 004-0041 Japan; 7 Department of Pharmacotherapy, Faculty of Pharmaceutical Sciences, Hokkaido University of Science, 15-4-1, Maeda 7, Teine-ku, Sapporo-shi, Hokkaido 006-8585 Japan

**Keywords:** Polymerase chain reaction, Antifungal stewardship, Candidemia, Rapid diagnostics, Antifungal agents

## Abstract

**Background:**

Candidemia has high mortality and requires early antifungal stewardship (AFS). Management by non-infectious disease specialists has been associated with worse outcomes, particularly in small- and medium-sized hospitals where infectious disease expertise is often limited. Rapid diagnostic tools such as polymerase chain reaction (PCR) may facilitate earlier stewardship support; however, their clinical impact in these settings remains unclear. This study aimed to evaluate the effect of PCR-based AFS on candidemia management in small- and medium-sized hospitals.

**Methods:**

We compared clinical data before and after the implementation of a PCR-based assay in patients aged ≥ 18 years with *Candida* species detected in blood cultures at Sapporo Tokushukai Hospital between June 2018 and May 2024 (*n* = 71 after exclusion). The primary endpoint was 30-day mortality rate. We also assessed the following secondary endpoints: rate of appropriate initial antifungal therapy, removal of central venous catheters (CVCs) within 24 h of diagnosis, prolonged antifungal therapy for ≥ 14 d after blood culture clearance, and time to antifungal therapy initiation.

**Results:**

No significant difference in 30-day mortality rate was observed between the pre-PCR and post-PCR groups (36.1% vs. 34.3%). Similarly, no significant differences were observed in rate of appropriate initial antifungal therapy, removal of CVCs within 24 h of diagnosis, prolonged antifungal therapy for ≥ 14 d after blood culture clearance, or time to therapy initiation. However, the proportion of physicians less familiar with candidemia care was significantly higher in the post-PCR group than in the pre-PCR group (68.6% vs. 36.1%, *P* = 0.01).

**Conclusions:**

PCR-based AFS might have allowed the quality of candidemia management and patient outcomes to be maintained, even in small- and medium-sized hospitals with a high proportion of physicians less familiar with candidemia care.

## Background

The mortality rate of candidemia is 35–67% [1–3], necessitating comprehensive management, including prompt administration of appropriate antifungal therapy and early screening for complications such as *Candida* endophthalmitis. The increasing resistance in *Candida* species to antifungal agents is a growing concern. Fluconazole (FLCZ) resistance in *Candida albicans* [4] and resistance associated with *FKS* gene mutations in *Candida glabrata* [5] have been reported, highlighting the importance of appropriate antifungal therapy in candidemia management.

In cases of septic shock secondary to candidemia, initiating effective antifungal therapy within 12–24 h of blood culture collection has been shown to reduce mortality [6]. Furthermore, antifungal stewardship (AFS), which encompasses measures such as the removal of central venous catheters (CVCs) within 24 h and screening for *Candida* endophthalmitis, is crucial for improving clinical outcomes [7–9]. AFS initiatives, widely implemented in large hospitals, have led to improvements in process indicators, such as a 26.8% reduction in total antifungal consumption and increased rates of appropriate antifungal use [10, 11], as well as in outcome indicators, including reduced 30-day mortality and shorter hospital stays [12]. However, evidence on AFS in small- and medium-sized hospitals remains scarce [13, 14], and its implementation varies depending on institutional and regional factors. A study of 240 hospitals in Japan found that only approximately 8% of facilities conducted interventions within 7 d for patients receiving antifungal therapy [15], highlighting challenges in the early implementation of AFS.

Polymerase chain reaction (PCR) is a nucleic acid amplification technique widely used for rapid identification of pathogens in infectious diseases. In the management of invasive fungal infections, PCR-guided AFS has been shown to shorten strain identification time, reduce the time to targeted therapy, lower antifungal agent costs, and decrease mortality [12, 16, 17]. Additionally, consultation by infectious disease specialists has been shown to improve the quality of candidemia management and patient outcomes [18, 19]. However, these reports are based on evidence from large hospitals, and it remains unclear whether PCR-based AFS can yield similar clinical outcomes in small- and medium-sized hospitals, where candidemia management is primarily overseen by physicians and healthcare professionals who are not specialized in infectious disease management. Investigating the clinical impact of PCR-based AFS in such hospitals could provide valuable insights. Therefore, this study aimed to examine the effect of PCR-based AFS on candidemia management in small- and medium-sized hospitals.

## Methods

### Facility overview

Sapporo Tokushukai Hospital is a small- and medium-sized hospital with 301 beds and a secondary emergency care function. No infectious disease specialists are involved in adult patient care. The microbiology laboratory is well-equipped, and positive blood culture reports are shared with the antimicrobial stewardship team (AST). AST physicians are responsible for general medical care. For this study, a dedicated pharmacist played a central role in selecting antifungal agents. This pharmacist is board-certified in both infection control and infectious disease chemotherapy. Since June 2021, PCR-based blood culture identification using the FilmArray^®^ Torch System (bioMérieux Japan Ltd., Tokyo, Japan) has been implemented. Antifungal susceptibility testing is outsourced only when requested by the attending physician, which is conducted only a few times per year, and its results typically take approximately 1 week to become available.

### Patients

All relevant patient information was obtained from medical records. This study included hospitalized patients (aged ≥ 18 years) at Sapporo Tokushukai Hospital with *Candida* spp. detected in blood cultures between June 2018 and May 2024. Patients were categorized into pre- (June 2018–May 2021) and post-PCR (June 2021–May 2024) groups, each spanning 3 years. Patients were excluded if they were transferred during treatment for candidemia, died before antifungal therapy, or had polymicrobial blood infections involving both bacteria and *Candida.*

### Microbiological testing

Blood cultures were incubated using a BACTEC FX System (Becton Dickinson [BD], Tokyo, Japan) in BD Bactec™ 23 F Aerobic Resin Bottle P and BD Bactec™ 22 F Anaerobic Resin Bottle P (both BD, Tokyo, Japan) for 7 d at 35 °C. Upon detection of a positive culture, the contents were directly subjected to genetic analysis using the FilmArray^®^ Torch System with the BioFire^®^ Blood Culture Panel (bioMérieux) and BioFire^®^ Blood Culture Panel 2 (bioMérieux). This molecular method was employed to ensure rapid identification and enhance the detection of polymicrobial infections, and it offers superior sensitivity compared with conventional culture methods. Subsequently, to confirm the genetic results and obtain isolates for further analysis, the positive culture bottles were subcultured onto Accurate™ Fractionated Sheep Blood Agar/Chocolate EX II (Shimadzu Diagnostics, Tokyo, Japan) under 5% CO₂, BD BBL™ BTB Lactose Agar (BD), and Vital Media Color Candida Agar (Kyokuto Pharmaceutical Industries, Tokyo, Japan) under aerobic conditions. The resulting isolates were identified using matrix-assisted laser desorption/ionization time-of-flight mass spectrometry (MALDI-TOF MS) using the MALDI Biotyper system (Bruker Daltonics, Kanagawa, Japan).

### Diagnostic and antifungal stewardship

The fundamental role of the dedicated pharmacist in AFS throughout the study periods included recommending appropriate antifungal therapy and promoting key clinical measures (CVCs removal, follow-up blood cultures, and continuation of antifungal therapy for ≥ 14 d after blood culture clearance). However, the timing of AFS differed. In the pre-PCR group, species-directed therapy and comprehensive stewardship were delayed until subculture and MALDI-TOF MS results became available, as initial notifications relied solely on Gram staining. In contrast, rapid pathogen identification in the post-PCR group enabled earlier AFS. After PCR results were obtained, the pharmacist promptly advised attending physicians on targeted therapy while recommending the standard clinical measures. The pharmacist’s effort devoted to the AST was 0.3 full-time equivalent (FTE) during the pre-PCR period, 0.7 FTE during the first 2 years of the post-PCR period (June 2021–May 2023), and 0.3 FTE during the final year.

### Primary and secondary endpoints

The primary endpoint was 30-day mortality rate [1, 20]. Secondary endpoints included time to mortality within 30 d, 90-day mortality rate [20, 21], intensive care unit (ICU) rate after therapy initiation, rate of appropriate initial antifungal therapy, rate of removal of CVCs within 24 h of diagnosis, rate of antifungal therapy for ≥ 14 d after blood culture clearance, cost of antifungal agents, and duration of antifungal therapy.

### Selection of initial antifungal therapy

Initial antifungal therapy was determined by attending physicians based on recommendations from the AST, considering the causative species and clinical severity, according to the Guidelines for the Management of Candidiasis [22, 23]. For critically ill patients, including those requiring ICU admission, MCFG was selected as first-line therapy. L-AMB was not routinely used as an initial option for all critically ill patients, although it was reserved for specific indications, including suspected *C. glabrata* endophthalmitis or insufficient response to other antifungal treatments.

### Definition of appropriate antifungal agents

Appropriate antifungal therapy selection was determined based on the *Candida* species involved and the presence of fungal endophthalmitis. In cases without fungal endophthalmitis, FLCZ or micafungin (MCFG) was considered appropriate for *Candida albicans*, *Candida tropicalis*, *Candida lusitaniae*, and *Candida dubliniensis*, whereas MCFG was used for *Candida glabrata* and FLCZ for *Candida parapsilosis*. In cases complicated by fungal endophthalmitis, liposomal amphotericin B (L-AMB) was administered for *Candida glabrata*, whereas FLCZ was considered appropriate for all other *Candida* species. Appropriate antifungal doses were recommended based on established guidelines [22, 23]. Specifically, a loading dose of 12 mg/kg followed by a maintenance dose of 6 mg/kg/d was recommended for FLCZ, and a daily dose of 100–150 mg was recommended for MCFG. For patients with an estimated glomerular filtration rate (eGFR) < 50 mL/min/1.73 m², the appropriate dose of FLCZ was defined as half the standard dose.

### Definitions and calculation methods for the study variables

The 30-day mortality rate and time to antifungal therapy initiation were determined from the date of positive blood culture submission. The rate of appropriate initial antifungal therapy was calculated based on the suitability of the antifungal agent selected for the identified *Candida* species. The duration of treatment was calculated from the initiation date of antifungal therapy. Antifungal therapy costs were calculated for all injectable antifungal agents used, based on the 2023 National Health Insurance drug price list in Japan. Costs were converted to U.S. dollars based on the average exchange rate during the study period (June 2018–May 2024; 122.9 JPY/USD). Unit prices of antifungal agents were as follows: FLCZ 100 mg, 1134 JPY; MCFG 100 mg, 1655 JPY; voriconazole 200 mg, 6493 JPY; and L-AMB 50 mg, 7647 JPY.

### Statistical analyses

Continuous variables for each endpoint are presented as medians with interquartile ranges and were compared between the pre- and post-PCR groups using the Student’s *t*-test for normally distributed variables and the Wilcoxon rank-sum test for non-normally distributed variables. Fisher’s exact test was used for categorical variables. To evaluate the time to initial antifungal therapy in the pre- and post-PCR groups, the proportion of initial antifungal therapy was plotted using the Kaplan–Meier method. The log-rank test was performed for intergroup comparisons. All statistical analyses were performed using JMP^®^ Pro 17.0.0 (JMP Statistical Discovery LLC, Cary, NC, USA), and a *P*-value of < 0.05 was considered statistically significant.

### Ethical considerations

This study was conducted in accordance with the Declaration of Helsinki and approved by the Institutional Review Board of the Mirai Medical Research Center, Inc. (Tokyo, Japan) (Ethical Review Number: TGE02452-010; approved on April 18, 2024). In accordance with the retrospective nature of the study, informed consent was not actively sought from individual patients; instead, information regarding an opt-out option was made publicly available via the hospital website, thereby ensuring participants had the opportunity to decline participation. All personal data were anonymized prior to analysis, and strict confidentiality was maintained throughout the study.

## Results

### Patient characteristics

**Fig. 1:**
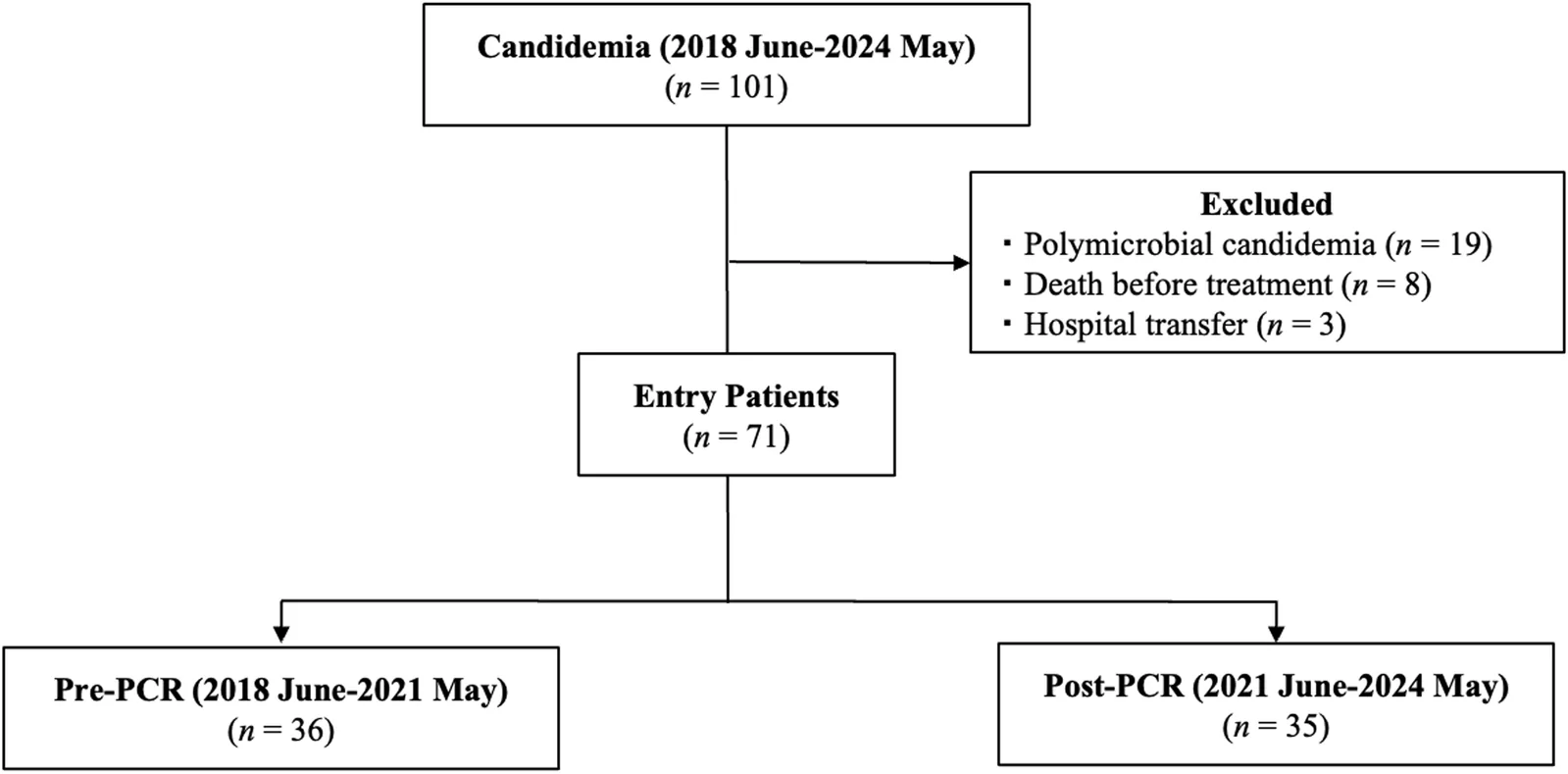
Flowchart depicting the screening process for eligible patients. In this study, 101 patients were recruited (between June 2018 and May 2024); 30 were excluded, and 71 were included in the analysis, with 36 assigned to the pre-PCR group and 35 to the post-PCR group. PCR, polymerase chain reaction

Figure 1 illustrates the target patient flow diagram. Of the 101 eligible patients, 30 were excluded based on the exclusion criteria: 19 had overlapping bacteremia with both bacteria and *Candida*, 8 died before antifungal treatment, and 3 were transferred. Consequently, 71 patients were included in the analysis. Patients were categorized into pre- (*n* = 36) and post-PCR (*n* = 35) groups. The annual mean number of *Candida* bloodstream infection cases was 14.3 pre-PCR and 19.3 post-PCR. The number of cases for each year from June 2018 to May 2024 was 11, 15, 17, 16, 15, and 27, respectively. Table 1 presents a comparison of patient characteristics between the two groups. Regarding patient characteristics, Charlson comorbidity index, ICU admission at therapy initiation, and Pitt candidemia score [24, 25] did not differ between the groups. The proportion of patients treated in departments without AST physicians was significantly higher in the post-PCR group than in the pre-PCR group (68.6% vs. 36.1%, *P* = 0.01). Regarding the selection of initial antifungal agents (FLCZ or MCFG), no significant difference was observed between the groups. No patient received L-AMB as initial therapy. The maintenance dose of FLCZ, adjusted according to renal function, was 6.9 (interquartile range 2.0–10.2) mg/kg in the pre-PCR group and 3.1 (2.3–4.3) mg/kg in the post-PCR group, and the median daily dose of MCFG was 150 (100–150) mg and 100 (100–100) mg, in the pre- and post-PCR groups, respectively; however, only the latter showed a significant difference (*P* = 0.13 and *P* = 0.02). No other significant differences in patient characteristics were observed between the groups. No patient had undergone hematopoietic stem cell transplantation or had hematologic malignancies. At blood culture collection, only one patient in the post-PCR group received prophylactic antifungal therapy, and no patient had β-D-glucan levels measured at treatment initiation.

**Table 1:** Patient characteristics

	Pre-PCR (*n* = 36)	Post-PCR (*n* = 35)	*P*-value
Male (%)	23 (63.9)	19 (54.3)	0.47
Age (IQR)	84.5 (72.5–89.0)	84.0 (79.0–91.0)	0.72
Body weight, kg (IQR)	44.2 (38.6–53.9)	47.0 (37.8–54.6)	0.78
Body surface area, m^2^ (IQR)	1.6 (1.5–1.6)	1.6 (1.5–1.7)	0.55
Charlson comorbidity index (IQR)	7 (6–8)	8 (7–9)	0.06
ICU at therapy initiation (%)	9 (25.0)	3 (8.6)	0.11
Pitt candidemia score (IQR)	3 (1–4)	2 (1–4)	1.00
Incidence in departments without AST physicians (%)	13 (36.1)	24 (68.6)	0.01**
Isolated *Candida* spp.†			0.87
* Candida albicans* (%)	19 (52.8)	16 (45.7)	
* Candida glabrata* (%)	10 (27.8)	13 (37.1)	
* Candida lusitaniae* (%)	1 (2.8)	1 (2.9)	
* Candida parapsilosis* (%)	5 (13.9)	4 (11.4)	
* Candida dubliniensis* (%)	0 (0)	1 (2.9)	
* Candida tropicalis* (%)	1 (2.8)	2 (5.7)	
Albumin, mg/dL (IQR)	2.4 (1.9–2.6)	2.2 (1.8–2.5)	0.59
T-Bil, mg/dL (IQR)	0.8 (0.5–1.0)	0.7 (0.5–1.2)	0.61
eGFR, mL/min/1.73 m^2^ (IQR)	68.9 (41.6–91.8)	45.1 (23.4–80.5)	0.06
CRP, mg/dL (IQR)	5.5 (2.7–11.7)	6.6 (2.9–10.8)	0.85
WBC, ×10^2^/uL (IQR)	86.0 (62.0–131.3)	79.0 (45.0–107.0)	0.35
Platelet, ×10^4^/uL (IQR)	15.6 (12.9–21.6)	16.1 (9.9–24.4)	0.86
Initial antifungal therapy			0.13
Fluconazole (%)	8 (22.2)	14 (40.0)	
Micafungin (%)	28 (77.8)	21 (60.0)	

Abbreviations: PCR, polymerase chain reaction; IQR, interquartile range; ICU, intensive care unit;

AST, antimicrobial stewardship team; T-Bil, total bilirubin; eGFR, estimated glomerular filtration rate; CRP, C-reactive protein; WBC, white blood cell

† Includes isolation of overlapping *Candida* spp. **P < 0.01

### Primary and secondary endpoints

Table 2 shows primary and secondary endpoints between the groups. The 30-day mortality rate was 36.1% in the pre-PCR group and 34.3% in the post-PCR group, without a significant difference. Regarding the secondary endpoints, the median time to mortality within 30 d was 7.0 and 9.0 d in the pre- and post-PCR groups (*P* = 0.20), respectively, and ICU admission after antifungal therapy initiation occurred in 7.4% and 0% of patients in the pre- and post-PCR groups (*P* = 0.22), respectively. The proportion of patients who received appropriate initial antifungal therapy was 80.6% and 91.4% in the pre- and post-PCR groups (*P* = 0.31), respectively. No significant differences were observed between the groups in removal of CVCs within 24 h of diagnosis or in prolonged antifungal therapy for ≥ 14 d after blood culture clearance.

**Table 2:** Primary and secondary endpoints

	Pre-PCR (*n* = 36)	Post-PCR (*n* = 35)	*P*-value
30-day mortality (%)	13 (36.1)	12 (34.3)	1.00
Time to mortality within 30 d, d (IQR)	7.0 (5.0–15.0)	9.0 (7.0–21.0)	0.20
90-day mortality (%)	16 (44.4)	19 (54.3)	0.48
ICU after therapy initiation (%)^†^	2/27 (7.4)	0/32 (0)	0.22
Rate of appropriate initial antifungal therapy (%)	29 (80.6)	32 (91.4)	0.31
Removal of existing CVCs within 24 h of diagnosis (%)	14/27 (51.9)	9/29 (31.0)	0.17
Antifungal therapy for ≥ 14 d after blood culture clearance (%)^††^	12/23 (52.2)	9/23 (39.1)	0.55
Cost of antifungal therapy, USD (IQR) ^†††^	1639.2 (615.5–2647.8)	1139.7 (516.8–1607.6)	0.06
Duration of antifungal therapy, d (IQR) ^††^	24.0 (17.0–57.0)	24.0 (16.0–35.0)	0.42

Abbreviations: PCR, polymerase chain reaction; IQR, interquartile range; ICU, intensive care unit; CVCs, central venous catheters

^†^The denominator excludes patients who were in the ICU at the time of therapy initiation

^††^Calculated excluding patients who died within 30 days

^†††^Calculated from all cases

### Time to antifungal therapy initiation

**Fig. 2:**
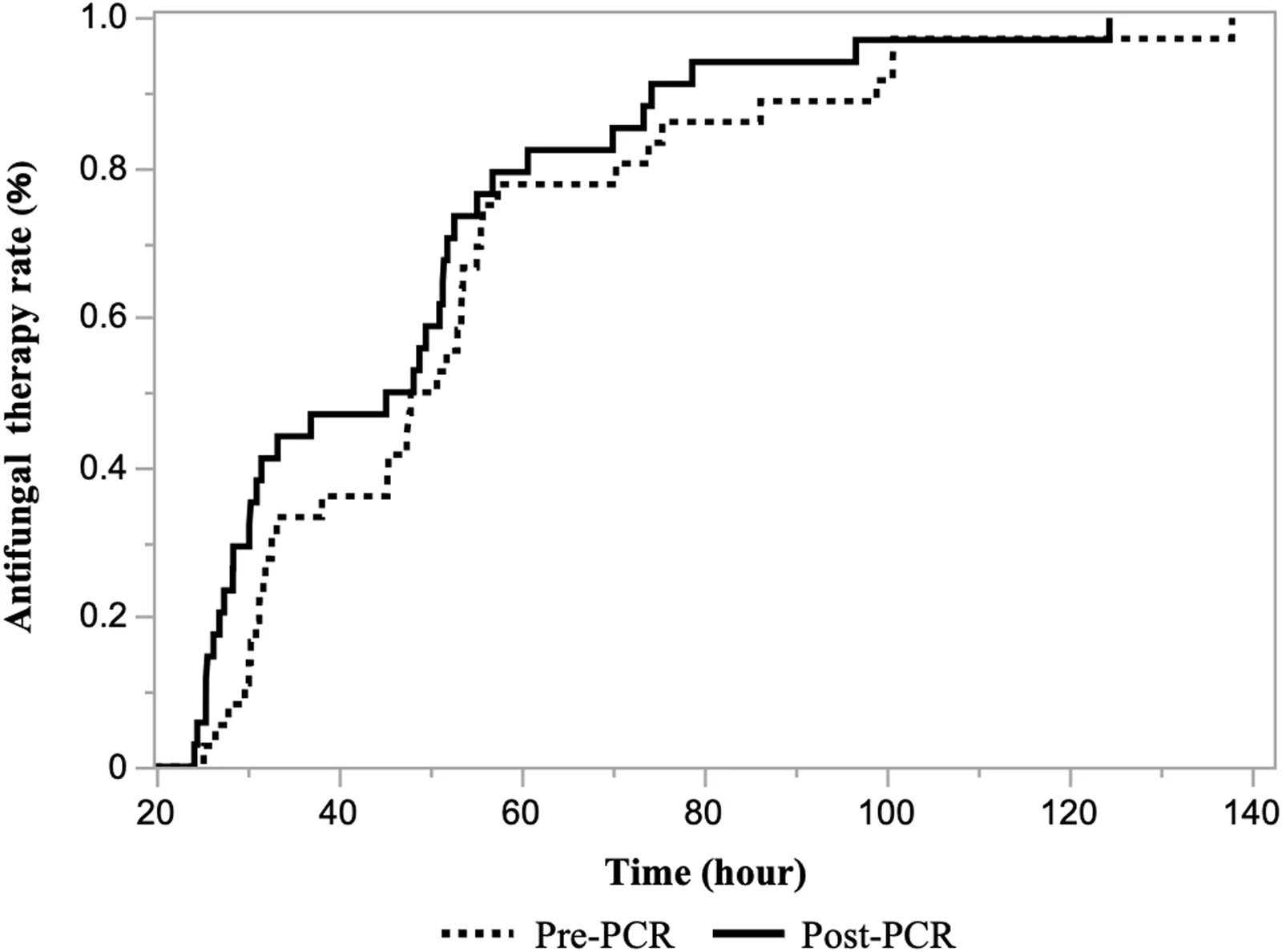
Log-rank test for time to antifungal therapy initiation. Abbreviations: PCR, polymerase chain reaction; 95% CI, 95% confidence interval. Dotted line: pre-PCR group. Solid line: post-PCR group. The median time to antifungal therapy initiation was 49.4 h (95% CI, 33.3–53.7) in the pre-PCR group and 46.8 h (95% CI, 30.3–51.6) in the post-PCR group, without significant differences (*P* = 0.23)

Figure 2 shows the results of the log-rank test for the time to antifungal therapy initiation. The median time to antifungal therapy initiation was 49.4 h (95% confidence interval [CI], 33.3–53.7) in the pre-PCR group and 46.8 h (95% CI, 30.3–51.6) in the post-PCR group (*P* = 0.23).

## Discussion

One of the key advantages of PCR is its ability to rapidly identify fungal species [17, 21, 26]. Previous studies [12, 17] have demonstrated that rapid fungal species identification contributes to shorter time to treatment and lower mortality. However, our study did not find a significant difference in 30-day mortality or in time to antifungal therapy initiation between the groups. Several factors may explain this discrepancy. At our institution, even in the pre-PCR period, we had a system in place to report Gram stain results from positive blood cultures to physicians on the same day, regardless of time. This allowed for early recognition of *Candida* bloodstream infections in both groups. In addition, according to our AFS survey, prior to PCR implementation, the median time to initiation of appropriate antifungal therapy was already around 48 h [14], as was the case in the pre-PCR group of the present study, which is earlier than the 59 h treatment delay in earlier reports [12]. Delayed antifungal therapy initiation increases the risk of mortality with each 24-h interval [27]. Therefore, we believe that the early therapy initiation at our institution prior to PCR implementation limited the additional benefit of PCR in shortening the time to treatment initiation, which may have influenced the 30-day mortality observed in this study. However, in small- and medium-sized hospitals, where microbiology laboratories are often outsourced and reporting is delayed, PCR may have a greater clinical impact by facilitating faster therapeutic decisions.

Consultation by infectious disease specialists has been shown to improve the quality of candidemia management and patient outcomes [18, 19]. In the post-PCR group, the proportion of cases managed by physicians outside AST was significantly greater compared with that in the pre-PCR group, which may have led to worse symptom management. However, there were no significant differences between the groups in mortality rates, time to antifungal therapy initiation, and key recommended management measures for candidemia (such as appropriate initial antifungal therapy, CVCs removal within 24 h, and appropriate treatment continuation after blood culture clearance), which was also confirmed in cases managed in departments without AST physicians (data not shown). Nonetheless, the tendency of shortened detection time and appropriate antifungal therapy was confirmed in the post-PCR group, maybe owing to less statistical power. None of the evaluated management indicators showed significant improvement following PCR implementation, and the overall effect of the management bundle could not be clearly demonstrated. However, the findings suggest that the quality of candidemia management and patient outcomes may have been maintained, or at least did not clearly deteriorate, despite a greater proportion of cases being managed by departments less familiar with candidemia care.

Regarding the rate of appropriate initial antifungal therapy, the proportion increased in the post-PCR group; however, the difference was not significant between the groups. The appropriateness of antifungal therapy is generally defined by the selection of agents according to *Candida* species and disease severity, as well as individualized dose optimization. The administered dose of MCFG was significantly lower in the post-PCR group than in the pre-PCR group. For *Candida* species, the ratio of the area under the blood concentration–time curve (AUC) to the minimum inhibitory concentration is associated with MCFG efficacy [28, 29]. Its AUC reduction has been a concern in patients in the ICU and in those with a body surface area (BSA) greater than 2.10 m² [28, 29]. However, the proportions of patients in the ICU after antifungal therapy and baseline Pitt candidemia [24, 25] score were similar between the groups, and no patient had a BSA exceeding 2.10 m², suggesting that differences in MCFG dose were unlikely to have affected the clinical outcomes.

This study had some limitations. First, it was a single-center retrospective study with a small sample size, which may have reduced the statistical power to detect meaningful differences and the generalizability of the results to clinical practice. In particular, the study may have been underpowered to detect differences in time to pathogen detection and appropriate antifungal selection. Second, the overall effect of the management bundle was also difficult to assess, preventing the demonstration of a clear clinical benefit. Third, owing to its pre–post design, the study lacked a contemporaneous control group, limiting the ability to draw definitive conclusions about the effectiveness of PCR implementation. Nevertheless, the findings offer preliminary evidence that may inform future research, despite potential confounding factors. Finally, the study included cases in which FLCZ dosing was not optimized, and antifungal susceptibility testing was not routinely performed. Given these limitations, the possibility that various unmeasured confounding factors or treatment-related biases have influenced the observed 30-day mortality outcomes cannot be excluded. To validate these findings, future research should include multicenter, controlled studies involving similarly scaled institutions that have not yet adopted PCR.

## Conclusions

The implementation of PCR-based AFS might have enabled early clinical decision-making in the management of candidemia. This approach may help maintain the quality of candidemia care and patient outcomes, even in small- and medium-sized hospitals—where the proportion of physicians unfamiliar with candidemia management may fluctuate over time.

## Data Availability

The datasets used and/or analyzed in the current study are available from the corresponding author upon reasonable request.

## Abbreviations

AFS: Antifungal stewardship
AST: Antimicrobial stewardship team
AUC: Area under the blood concentration time curve
BSA: Body surface area
C. albicans: Candida albicans
C. dubliniensis: Candida dubliniensis
C. glabrata: Candida glabrata
C. lusitaniae: Candida lusitaniae
C. parapsilosis: Candida parapsilosis
C. tropicalis: Candida tropicalis
CI: Confidence interval
CVCs: Central venous catheters
eGFR: Estimated glomerular filtration rate
FLCZ: Fluconazole
FTE: Full-time equivalent
ICU: Intensive care unit
L-AMB: liposomal amphotericin B
MCFG: Micafungin
PCR: Polymerase chain reaction
T-Bil: Total bilirubin
